# Systemic Inflammation and Outcome in 2295 Patients with Stage I–III Colorectal Cancer from Scotland and Norway: First Results from the ScotScan Colorectal Cancer Group

**DOI:** 10.1245/s10434-020-08268-1

**Published:** 2020-04-04

**Authors:** James H. Park, Anniken J. Fuglestad, Anne H. Køstner, Agata Oliwa, Janet Graham, Paul G. Horgan, Campbell S. D. Roxburgh, Christian Kersten, Donald C. McMillan

**Affiliations:** 1 Academic Unit of Surgery, School of Medicine Dentistry and Nursing, College of Medicine, Veterinary and Life Sciences, University of Glasgow, Glasgow, UK; 2 Center for Cancer Treatment, Sørlandet Hospital, Kristiansand, Norway; 3 Institute of Cancer Sciences, College of Medicine, Veterinary and Life Sciences, University of Glasgow, Glasgow, UK

## Abstract

**Background:**

Systemic inflammatory response (SIR) is an adverse prognostic marker in colorectal cancer (CRC) patients. The ScotScan Colorectal Cancer Group was established to examine how markers of the SIR differ between populations and may be utilised to guide prognosis.

**Patients and Methods:**

Patients undergoing resection of stage I–III CRC from two prospective datasets in Scotland and Norway were included. The relationship between the modified Glasgow Prognostic Score (mGPS; combination of C-reactive protein and albumin) and overall survival (OS) was examined. The relationship between OS, adjuvant chemotherapy regime and mGPS was examined in patients with stage III colon cancer.

**Results:**

A total of 2295 patients were included. Patients from Scotland were more inflamed despite controlling for associated characteristics using multivariate logistic regression or propensity score matching (OR 2.82, 95% CI 1.98–4.01, *p *< 0.001). mGPS had similar independent prognostic value in both cohorts (Scotland: HR 1.27, 95% CI 1.12–1.45; Norway: HR 1.23, 95% CI 1.01–1.49) and stratified survival independent of TNM group in the whole cohort. In patients with stage III colon cancer receiving adjuvant therapy, there appeared to be a survival benefit in systemically inflamed patients receiving oxaliplatin but not single-agent 5-fluorouracil or capecitabine.

**Conclusions:**

The SIR differs between populations from different countries; however prognostic value remains similar. The present study strongly supports the routine reporting of the mGPS in patients with CRC.

**Electronic supplementary material:**

The online version of this article (10.1245/s10434-020-08268-1) contains supplementary material, which is available to authorized users.

Systemic inflammation is an important determinant of disease progression and outcome in patients with cancer.^1^ The body of evidence supporting the routine assessment of indices of the systemic inflammatory response (SIR) as prognostic markers is such that recent consensus statements have proposed their mandatory inclusion in future oncology trials of patients with advanced colorectal and pancreatic cancer.^2^^,^^3^

Similarly, markers of the SIR may also inform prognosis of patients undergoing potentially curative treatment. In this regard, two recent meta-analyses have confirmed the independent prognostic value of the modified Glasgow Prognostic Score (mGPS), a cumulative score based on circulating serum C-reactive protein (CRP) and albumin concentrations, in patients with primary operable colorectal cancer.^4^^,^^5^ A model by which the combination of TNM stage and mGPS could be utilised to determine prognosis following surgical resection in patients with stage I–III disease has previously been proposed.^6^ Using such a scheme provides further risk stratification than either measure alone; for example, whereas 5-year cancer-specific survival of patients with stage III colon cancer overall was 63%, the addition of the mGPS further stratified survival from 75 to 37%.

However, although of use in determining prognosis, whether the systemic inflammatory response may also aid in the selection of patients for adjuvant therapy remains to be determined. Both observational studies and clinical trials have confirmed that the mGPS and related scores retain prognostic significance in patients receiving chemotherapy and radiotherapy.^6^^–^8 Whether this represents a need to select the appropriate chemotherapy regime and duration on the basis of the SIR or simply reflects futility of conventional cytotoxic chemotherapy in the presence of systemic inflammation remains unclear.

The ScotScan Collaborative was established by two multidisciplinary groups from Glasgow, United Kingdom, and Southern Hospital Trust, Norway, with a mutual interest in the role of host inflammatory responses in determining oncological outcomes in patients with colorectal cancer. It was perceived that the combined resources of these two groups could be utilised to address a number of unanswered questions and further refine the clinical application of inflammatory-based measures to determine prognosis and treatment strategies of patients with colorectal cancer. In the present study, the ScotScan Collaborative dataset is described and the relationships between mGPS and survival are reported. Furthermore, this combined dataset is used to further examine the relationship between mGPS, adjuvant therapy and survival of patients with stage III colon cancer.

## Patients and Methods

### Scotish and Norwegian Cohort

Patients were identified from a prospectively maintained database of colorectal cancer resections performed at Glasgow Royal Infirmary since January 1997 and at Southern Hospital Trust in Norway since January 2000. For the present work, patients who underwent resection of TNM stage I–III colorectal adenocarcinoma between January 1997 and June 2015 in Scotland and between January 2000 and May 2017 in Norway with curative intent (based on pre-operative cross-sectional imaging and intra-operative findings) were included. Both elective and emergency cases were included. Patients who underwent palliative or localised resection and those who did not have pre-operative measurement of CRP were excluded.

Serum albumin and CRP were measured at pre-operative assessment within 30 days of surgery for elective patients and on day of admission for patients undergoing emergency surgery. The mGPS was calculated as previously described; 9 patients with CRP ≤ 10 mg/L were allocated a score of 0; patients with CRP > 10 mg/L alone were allocated a score of 1; and patients with CRP > 10 mg/L and albumin < 35 g/L a score of 2. Pathological staging of tumours was performed using TNM fifth edition for patients from Scotland, consistent with contemporary reporting guidelines during the time period studied.^10^ In Norway, the fifth TNM edition was used until January 2009, seventh edition until August 2017 and eighth edition afterwards. Tumours were classified as right (caecum to distal transverse), left (splenic flexure to rectosigmoid) and rectal (distal to rectosigmoid).

All patients were discussed at weekly colorectal cancer multidisciplinary meetings prior to and following surgery. Those with both node-positive disease and node-negative disease with high-risk characteristics (i.e. T4, perforation, venous invasion) were considered for systemic adjuvant chemotherapy. Patients were routinely followed up for 5 years, according to local institutional guidelines. Date and cause of death were confirmed using hospital electronic case records; follow-up and confirmation of vital status were censored on 30 June 2017 for patients from Scotland. Date of last recorded follow-up or last review of electronic case records (31 December 2017) acted as the censor date for patients from Norway. Overall survival was measured from date of surgery for Scotland and first confirmed biopsy (including surgery) for Norway, until date of death from any cause. Cancer-specific survival was measured until date of death from radiologically or histologically confirmed recurrent colorectal cancer. Local institutional ethics approval was obtained from both hospitals.

### Statistics

Categorical data were examined using *χ*^2^ analysis for linear trend, and the relationship between clinicopathological characteristics and the mGPS was examined using binary logistic regression to calculate odds ratios (OR) and 95% confidence intervals (CI). To test for independence, a multivariate backwards conditional model was constructed using variables with *p *< 0.05 on univariate analysis. To further account for differences in clinical and pathological characteristics associated with the two different cohorts, propensity score matching was also performed using the following variables: age, American Society of Anesthesiologists (ASA) grade, presentation, neoadjuvant therapy, tumour location, T stage, N stage and differentiation.

The relationship between clinicopathological characteristics and overall survival and cancer-specific survival was examined using Cox proportional hazards regression to calculate hazard ratios (HR) and 95% CI. Multivariate survival analysis was performed using a backwards conditional method, including variables with *p *< 0.05 on univariate analysis. To account for differences in treatment over the time period studied, year of surgery was divided into quartiles and entered as a variable into all multivariate models. Three-year overall survival was reported as percentage surviving [standard error (SE)] and displayed using Kaplan–Meier curves, with log-rank survival analysis to compare survival between groups. A *p* value < 0.05 was considered statistically significant. All analyses were performed using SPSS version 25 for Mac (IBM SPSS, Armonk NY, USA).

## Results

### The ScotScan Cohort

The clinicopathological characteristics of 2295 patients who underwent resection of stage I–III colorectal cancer in Scotland (*n *= 1234) and in Norway (*n *= 1061) are displayed in Table 1. Patients from Norway were more likely to be older, female and have more comorbidity (*p *< 0.001). Patients from Scotland were more likely to have had surgery for rectal cancer and more advanced TNM stage. Emergency presentation and neoadjuvant chemoradiotherapy use were comparable between groups, however patients from Scotland were more likely overall to receive adjuvant chemotherapy. When categorised by stage, patients from Scotland were more likely to receive adjuvant therapy for stage II disease (17% versus 2%, *p *< 0.001), whereas those from Norway were more likely to receive adjuvant therapy for stage III disease (54% versus 47%, *p *< 0.001). Patients from Scotland were more likely to be systemically inflamed prior to surgery as measured by both CRP > 10 mg/L and mGPS (both *p *< 0.001).

**Table 1:** Comparison of clinicopathological characteristics of patients from Scotland and Norway undergoing potentially curative resection of stage I–III colorectal cancer

Clinicopathological characteristics		Scotland	Norway	*p*
		(*N *= 1234) (%)	(*N *= 1061) (%)	
Age (years)				< 0.001
	< 65	420 (34)	248 (23)	
	65–74	431 (35)	327 (31)	
	> 75	383 (31)	486 (46)	
Sex				< 0.001
	Female	547 (44)	562 (53)	
	Male	687 (56)	499 (47)	
ASA grade (1883)				< 0.001
	I	167 (19)	43 (4)	
	II	366 (42)	411 (41)	
	III	310 (35)	505 (50)	
	IV	39 (4)	42 (4)	
Presentation (2294)				0.497
	Elective	1118 (91)	969 (91)	
	Emergency	116 (9)	91 (9)	
Year of surgery				< 0.001
	1997–2005	454 (37)	58 (6)	
	2006–2010	297 (24)	223 (21)	
	2011–2013	319 (26)	254 (24)	
	2014–2017	164 (13)	526 (49)	
Neoadjuvant therapy (2287)				0.634
	No	1102 (90)	960 (90)	
	Yes	124 (10)	101 (10)	
Adjuvant chemotherapy (2248)				< 0.001
	No	279 (74)	904 (85)	
	Yes	308 (26)	157 (15)	
Tumour subsite				< 0.001
	Right	449 (37)	464 (44)	
	Left	376 (31)	339 (32)	
	Rectum	403 (33)	258 (24)	
T stage				< 0.001
	0	16 (1)	13 (1)	
	1	93 (7)	101 (10)	
	2	142 (12)	201 (19)	
	3	677 (55)	691 (65)	
	4	306 (25)	55 (5)	
N stage				0.001
	0	767 (62)	722 (68)	
	1	330 (27)	255 (24)	
	2	137 (11)	84 (8)	
TNM stage				< 0.001
	PCR	16 (1)	12 (1)	
	I	196 (16)	259 (24)	
	II	555 (45)	451 (43)	
	III	467 (38)	339 (32)	
Differentiation (2204)				< 0.001
	Well/mod	1103 (91)	839 (85)	
	Poor	115 (9)	147 (15)	
C-reactive protein				0.001
	≤ 10 mg/L	807 (65)	761 (72)	
	> 10 mg/L	427 (35)	300 (28)	
Albumin				< 0.001
	≥ 35 g/L	900 (73)	937 (88)	
	< 35 g/L	334 (27)	124 (12)	
mGPS				< 0.001
	0	807 (65)	761 (72)	
	1	231 (19)	201 (19)	
	2	196 (16)	99 (9)	

(*n*) given when incomplete data available. *p* value given for *χ*^2^ method for linear trend for categorical variables

### Relationship Between Clinicopathological Characteristics and mGPS

Differences in SIR between the two cohorts were examined using both the whole cohort and a propensity score matched cohort. On univariate binary logistic regression analysis (Table 2), advancing age and ASA grade, emergency presentation, advancing T and N stage and poor tumour differentiation were all associated with an elevated mGPS, whereas male sex, Norwegian cohort, year of surgery quartile, neoadjuvant therapy and rectal primary were associated with lower risk of an elevated mGPS. On multivariate analysis, male sex (OR 0.74, *p *= 0.007), Norwegian cohort (OR 0.65, *p *< 0.001), high ASA grade (OR 1.32, *p *< 0.001), year of surgery (OR 0.75, *p *< 0.001), emergency presentation (OR 4.34, *p *< 0.001), distal primary location (OR 0.72, *p *< 0.001), advancing T stage (OR 2.13, *p *< 0.001) and poor differentiation (OR 2.04, *p *< 0.001) were all independently associated with mGPS.

**Table 2:** Relationship between clinicopathological characteristics and presence of elevated systemic inflammatory responses (mGPS ≥ 1) in patients undergoing potentially curative resection of stage I–III colorectal cancer in Norway and Scotland

	Univariate OR (95% CI)	*p*	Multivariate OR (95% CI)	*p*
*Full cohort * (*N *= 2295)				
Age (< 65/65–74/> 74)	1.23 (1.10–1.37)	< 0.001	–	0.317
Sex (female/male)	0.76 (0.63–0.90)	0.002	0.75 (0.60–0.94)	0.012
Centre (Scotland/Norway)	0.75 (0.62–0.89)	0.001	–	0.321
ASA grade (I/II/III/IV)	1.40 (1.23–1.61)	< 0.001	1.25 (1.07–1.45)	0.004
Year of surgery quartile	0.73 (0.67–0.79)	< 0.001	0.75 (0.68–0.83)	< 0.001
Presentation (elective/emergency)	6.20 (4.53–8.49)	< 0.001	4.58 (3.09–6.77)	< 0.001
Neoadjuvant therapy (no/yes)	0.34 (0.23–0.50)	< 0.001	–	0.213
Tumour site (right/left/rectum)	0.59 (0.53–0.66)	< 0.001	0.71 (0.61–0.82)	< 0.001
T stage (0/1/2/3/4)	2.58 (2.24–2.97)	< 0.001	2.16 (1.80–2.60)	< 0.001
N stage (0/1/2)	1.26 (1.11–1.44)	< 0.001	–	0.096
Differentiation (mod-well/poor)	2.76 (2.13–3.59)	< 0.001	2.11 (1.53–2.90)	< 0.001
*Colon cancer without neoadjuvant therapy* (*N *= 1618)				
Age (< 65/65–74/> 74)	1.17 (1.03–1.32)	0.016	–	0.239
Sex (female/male)	0.79 (0.64–0.96)	0.020	0.73 (0.57–0.93)	0.013
Centre (Scotland/Norway)	0.79 (0.64–0.96)	0.019	–	0.835
ASA grade (I/II/III/IV)	1.30 (1.11–1.51)	0.001	1.21 (1.02–1.44)	0.025
Year of surgery quartile	0.74 (0.67–0.80)	< 0.001	0.78 (0.70–0.87)	< 0.001
Presentation (elective/emergency)	4.91 (3.55–6.79)	< 0.001	4.13 (2.78–6.14)	< 0.001
Tumour site (right colon/left colon)	0.72 (0.59–0.89)	0.002	–	0.090
T stage (0/1/2/3/4)	2.55 (2.16–3.02)	< 0.001	2.01 (1.64–2.47)	< 0.001
N stage (0/1/2)	1.27 (1.10–1.48)	0.001	–	0.231
Differentiation (mod-well/poor)	2.84 (2.12–3.81)	< 0.001	2.25 (1.59–3.19)	< 0.001

*HR* hazard ratio, *CI* confidence interval, *ASA* American Society of Anesthesiologists

Propensity score matching was performed to match the two cohorts (Supplementary Fig. 1); despite close matching on the basis of stage and clinical characteristics (*n *= 736, Supplementary Table 1), patients from Scotland remained more likely to be systemically inflamed prior to surgery (mGPS ≥ 1, OR 2.82, 95% CI 1.98–4.01, *p *< 0.001). When patients were stratified by year of surgery, those from Scotland again remained more likely to be systemically inflamed (data not shown).

To account for differences in treatment modalities in patients with rectal cancer, further analysis was performed in the unmatched cohort, including only patients with colon cancer undergoing surgery without prior neoadjuvant treatment (*n *= 1618). Patient demographics reflected those of the original cohort (Supplementary Table 2). Patients from Scotland remained more likely to be systemically inflamed; on multivariate analysis, sex, country cohort, ASA grade, year of surgery, emergency presentation, T stage and differentiation all remained independently associated with mGPS (Table 2).

### Survival

Thirty-day mortality was 2% (54 patients); these patients were excluded from survival analysis (*n *= 2241). Median follow-up of survivors from Scotland was 70 months (interquartile range 45–120), with 300 cancer-associated and 264 non-cancer deaths; median follow-up of survivors from Norway was 29 months (8–51), with 94 and 137 cancer-associated and non-cancer deaths, respectively.

On both univariate and multivariate analysis, the mGPS had comparable prognostic value for overall survival in both patient cohorts (Supplementary Table 3; Scotland: multivariate HR 1.27, 95% CI 1.12–1.45, *p *< 0.001; Norway: multivariate HR 1.23, 95% CI 1.01–1.49, *p *= 0.043); therefore, further survival analysis was performed on the combined cohort.

On multivariate survival analysis (Table 3), mGPS remained associated with overall survival (HR 1.28, 95% CI 1.15–1.43, *p *< 0.001) independent of age, ASA grade, year of surgery quartile, adjuvant therapy, T stage and N stage, and with cancer-specfic survival (HR 1.36, 95% CI 1.15–1.61, *p *< 0.001) independent of ASA grade, year of surgery, T stage and N stage. Furthermore, the mGPS remained independently associated with survival when analysis was repeated in patients with colon cancer only (*n *= 1579, overall survival: HR 1.21, 95% CI 1.06–1.39, *p *= 0.005; cancer-specific survival: HR 1.26, 95% CI 1.03–1.55, *p *= 0.025) and when emergency patients were excluded (*n *= 1393, overall survival: HR 1.26, 95% CI 1.10–1.45, *p *= 0.001; cancer-specific survival: HR 1.29, 95% CI 1.03–1.621, *p *= 0.025). To account for differences in ascertainment of survival data between cohorts, only overall survival was examined in further analyses.

**Table 3:** Relationship between clinicopathological characteristics and overall survival of patients undergoing potentially curative resection of stage I–III colorectal cancer

	Overall survival				Cancer-specific survival			
	Univariate HR (95% CI)	*p*	Multivariate HR (95% CI)	*p*	Univariate HR (95% CI)	*p*	Multivariate HR (95% CI)	*p*
*Full cohort * (*N *= 2241)								
Age (< 65/65–74/> 74)	1.82 (1.65–2.00)	< 0.001	1.48 (1.31–1.67)	< 0.001	1.22 (1.08–1.39)	0.002	–	0.610
Sex (female/male)	1.12 (0.97–1.29)	0.132	–	–	1.21 (0.99–1.50)	0.067	–	0.075
ASA grade (I/II/III/IV)	2.11 (1.87–2.38)	< 0.001	1.72 (1.50–1.96)	< 0.001	1.65 (1.39–1.96)	< 0.001	1.45 (1.21–1.74)	< 0.001
Presentation (elective/emergency)	1.64 (1.31–2.04)	< 0.001	–	0.179	2.44 (1.85–3.22)	< 0.001	–	0.081
Year of surgery quartile	0.78 (0.72–0.85)	< 0.001	0.88 (0.80–0.98)	0.015	0.69 (0.61–0.77)	< 0.001	0.82 (.71–0.94)	0.004
Neoadjuvant therapy (no/yes)	0.68 (0.51–0.90)	0.007	–	0.724	1.03 (0.74–1.44)	0.853	–	–
Adjuvant chemotherapy (No/yes)	0.73 (0.60–0.88)	0.001	0.71 (0.55–0.91)	0.008	1.22 (0.97-–1.55)	0.093	–	0.054
Tumour site (right/left/rectum)	0.88 (0.81–0.96)	0.004	–	0.281	1.03 (0.91–1.17)	0.642	–	–
T stage (0/1/2/3/4)	1.51 (1.37–1.66)	< 0.001	1.31 (1.15–1.49)	< 0.001	2.35 (2.00–2.75)	< 0.001	1.92 (1.55–2.38)	< 0.001
N stage (0/1/2)	1.44 (1.31–1.59)	< 0.001	1.50 (1.32–1.71)	< 0.001	2.00 (1.75–2.28)	< 0.001	1.77 (1.49–2.11)	< 0001
Differentiation (mod-well/poor)	1.37 (1.11–1.70)	0.003	–	0.634	1.46 (1.08–1.97)	0.013	–	0.922
Modified Glasgow Prognostic Score (0/1/2)	1.54 (1.41–1.69)	< 0.001	1.28 (1.15–1.43)	< 0.001	1.67 (1.47–1.89)	< 0.001	1.36 (1.15–1.61)	< 0.001
*Colon cancer without neoadjuvant* (*N *= 1579)								
Age (< 65/65–74/> 74)	1.94 (1.73–2.17)	< 0.001	1.65 (1.42–1.92)	< 0.001	1.29 (1.10–1.51)	0.002	–	0.066
Sex (female/male)	1.00 (0.84–1.18)	0.975	–	–	1.12 (0.87–1.44)	0.388	–	–
ASA grade (I/II/III/IV)	2.28 (1.97–2.64)	< 0.001	1.78 (1.51–2.09)	< 0.001	1.79 (1.45–2.23)	< 0.001	1.51 (1.21–1.89)	< 0.001
Presentation (elective/emergency)	1.66 (1.32–2.09)	< 0.001	1.35 (1.01–1.81)	0.042	2.74 (2.04–3.67)	< 0.001	1.65 (1.13–2.39)	0.009
Year of surgery quartile	0.80 (0.72–0.88)	< 0.001	0.89 (0.79–1.00)	0.051	0.68 (0.59–0.78)	< 0.001	0.81 (0.69–0.95)	0.010
Adjuvant therapy (no/yes)	0.62 (0.49–0.77)	< 0.001	0.69 (0.51–0.94)	0.017	1.15 (0.866–1.53)	0.334	–	–
Tumour site (right colon/left colon)	0.84 (0.71–0.99)	0.043	–	0.540	0.95 (0.74–1.22)	0.686	–	–
T stage (0/1/2/3/4)	1.50 (1.33–1.69)	< 0.001	1.28 (1.09–1.50)	0.003	2.80 (2.27–3.46)	< 0.001	2.31 (1.75–3.04)	< 0.001
N stage (0/1/2)	1.39 (1.23–1.56)	< 0.001	1.45 (1.25–1.69)	< 0.001	1.99 (1.70–2.34)	< 0.001	1.65 (1.36–2.01)	< 0.001
Differentiation (mod-well/poor)	1.20 (0.94–1.53)	0.142	–	–	1.14 (0.79–1.64)	0.497	–	–
Modified Glasgow Prognostic Score (0/1/2)	1.55 (1.39–1.72)	< 0.001	1.21 (1.06–1.39)	0.005	1.68 (1.45–1.96)	< 0.001	1.26 (1.03–1.55)	0.025

*HR* hazard ratio, *CI* confidence interval, *ASA* American Society of Anesthesiologists

**Fig. 1:**
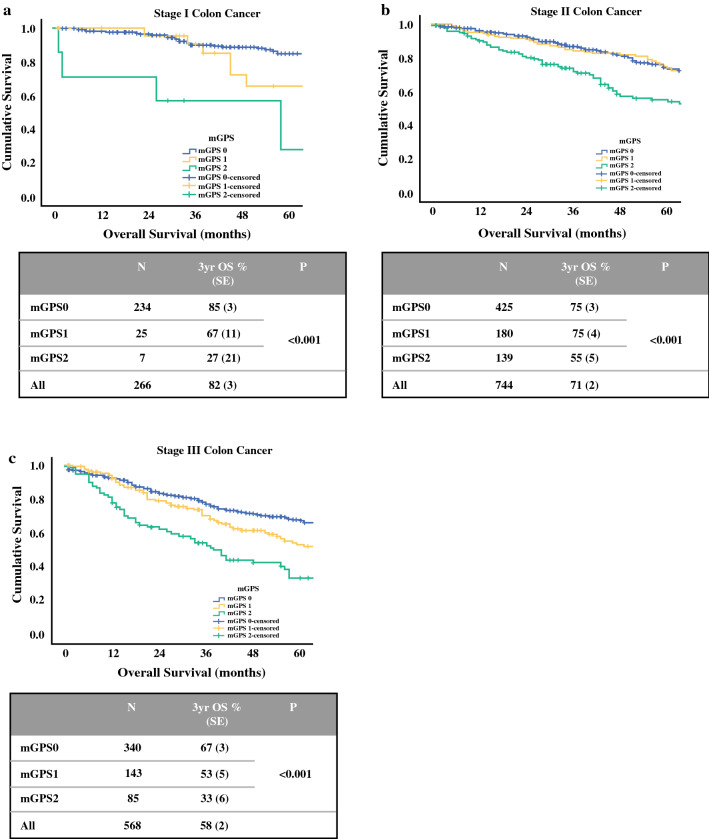
Relationship between modified Glasgow Prognostic Score and overall survival of patients undergoing resection of **a** stage I, **b** stage II and **c** stage III colon cancer in Scotland and Norway

The relationship between TNM stage, mGPS and 3-year overall survival of patients undergoing resection of colon cancer was further examined (Fig. 1). Overall 3-year survival of the combined cohort was 73%; TNM stratified survival from 82% (TNM I) to 58% (TNM III), whereas mGPS stratified survival from 74 to 46% (both *p *< 0.001). When combined, mGPS was able to stratify survival within TNM stage; for example 3-year overall survival of patients with stage I colon cancer was 85% (mGPS 0, *n *= 234), 67% (mGPS 1, *n *= 25) and 27% (mGPS 2, *n *= 7). Similarly, 3-year survival of patients with stage III disease was 67% (mGPS 0, *n *= 340), 53% (mGPS 1, *n *= 143) and 33% (mGPS 2, *n *= 85).

Subgroup analysis of patients undergoing elective resection of rectal cancer (*n *= 425) without neoadjuvant chemoradiotherapy was performed. The mGPS was associated with increasing T stage (*p *< 0.001) but no other clinical or pathological characteristics (data not shown). Small numbers within individual TNM/mGPS groups precluded meaningful analysis by stage, however the mGPS stratified 3-year overall survival from 86 to 76% (*p *= 0.009), and cancer-specific survival from 90 to 83% (*p *= 0.03).

Subgroup analysis of patients undergoing emergency resection (*n* = 207) was performed. Over 95% of patients underwent surgery for a T3/4 tumour, and 49% had node positive disease; 71% of patients were systemically inflamed at time of surgery; neither overall (*p *= 0.546) nor cancer-specific survival (*p *= 0.219) differed significantly between groups. Patient and tumour characteristics and use of adjuvant therapy were not associated with pre-operative systemic inflammatory response in those patients undergoing emergency resection (data not shown).

### Systemic Inflammation, Adjuvant Therapy and Overall Survival

**Fig. 2:**
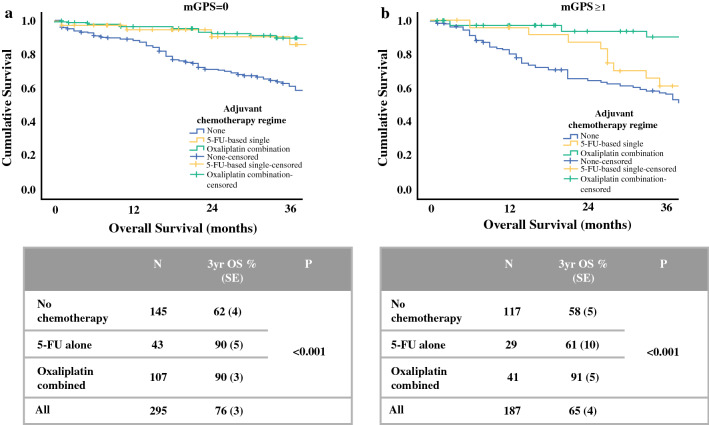
Relationship between chemotherapy regime and overall survival of patients with stage III colon cancer and **a** modified Glasgow Prognostic Score = 0 and **b** modified Glasgow Prognostic Score ≥ 1

Survival was examined in 482 patients with stage III colon cancer in whom adjuvant chemotherapy status was known (Fig. 2). Patients with stage III colon cancer in whom chemotherapy data were missing were more likely to be younger and male but did not differ with respect to T stage or mGPS (data not shown). For the purposes of further analysis, systemic inflammatory status was categorised as mGPS = 0 or mGPS ≥ 1, with 3-year overall survival of 76% and 65%, respectively (*p *= 0.01). Chemotherapy status was categorised as no chemotherapy (*n *= 262), 5-fluorouracil-based (5-FU) single-agent therapy (either infusional 5-FU or oral capecitabine, *n *= 72) or oxaliplatin-based combination therapy (with either oral or infusional 5-FU, *n *= 148), with 3-year survival of 60%, 76% and 90%, respectively (*p *< 0.001).

In patients with mGPS = 0, 5-FU and oxaliplatin-based chemotherapy were both associated with improved survival compared with patients not receiving adjuvant therapy (*p *< 0.001). In patients with mGPS ≥ 1 however, only oxaliplatin-based chemotherapy was associated with improved survival compared with no treatment (*p *< 0.001), with 3-year survival comparable to non-inflamed patients receiving either 5-FU or oxaliplatin. In contrast, patients with mGPS ≥ 1 receiving 5-FU only had no better 3-year overall survival than those without adjuvant chemotherapy.

The clinicopathological characteristics of patients receiving adjuvant chemotherapy were examined (Supplementary Table 4). Of patients receiving 5-FU alone, those with an elevated CRP were more likely to have been operated on during an earlier time period, have a higher T stage (both *p *< 0.05) and show a trend towards emergency presentation and poor differentiation. Of those patients receiving combined oxaliplatin therapy, an elevated CRP was associated with emergency presentation, T stage and differentiation (all *p *≤ 0.001) and showed a trend towards more proximal tumour location. When comparison was made between patients with an elevated CRP receiving different chemotherapy regimens, patients receiving oxaliplatin combination therapy were more likely to be younger (*p *< 0.001) and show a trend towards lower ASA grade (*p *= 0.091).

## Discussion

Utilising a dataset of over 2000 patients from two Northern European countries, the present study is, to the best of the authors’ knowledge, the largest prospective dataset in operable stage I–III CRC to date examining the systemic inflammatory response and outcome, and further confirms the strong prognostic value of the mGPS independent of disease stage.

The proportion of patients exhibiting elevated systemic inflammatory responses differed between the two populations, with patients from Scotland more likely to be systemically inflamed. These differences persisted even after controlling for clinical and pathological factors known to be associated with the systemic inflammatory response. Previous studies comparing differing populations with colorectal cancer have suggested that ethnicity is associated with systemic inflammatory responses prior to surgery.^11^ However, the two populations presently studied are Northern European, with both hospitals serving regions with predominantly Caucasian populations. In addition, both ancestry studies and disease susceptibility studies have suggested genetic homogeneity between Scottish and Scandinavian populations.^12^^–^15 Therefore, this would be unlikely to account significantly for the differences observed.

Differences in systemic inflammatory responses may reflect differences in clinical characteristics and tumour pathology; for instance increasing age and co-morbidity both more common in the Norwegian cohort, have previously been associated with the systemic inflammatory response in cancer.^14^^,^^15^ Similarly, pathological characteristics, such as advanced T stage, are associated with the mGPS and other inflammatory indices.^15^^,^^16^ Despite these unfavourable characteristics for the Norwegian cohort, patients from Scotland still exhibited more inflammation than the Norwegian patients, even after controlling for such variables using both multivariate adjustment and propensity score matching.

Similarly, the proportion of systemically inflamed patients decreased in association with year of surgery in both cohorts. The reason for this is not clear, however may represent a change in the characteristics of patients undergoing surgery for colorectal cancer; For instance screening was introduced in Scotland 2007–2009, with patients with screen-detected cancer less likely to have advanced disease stage at presentation.^17^ Similarly, optimisation of other medical comorbidities in more recently diagnosed patients may also impact upon the presence of a systemic inflammatory response. Given that 61% of patients from Glasgow Royal Infirmary (GRI) underwent surgery prior to 2011 compared with 27% of patients from Norway, this may in part explain differences in the proportion of systemically inflamed patients in each of the two cohorts studied. However, country of origin remained an independent determinant of the systemic inflammatory response even after controlling for year of surgery.

The differences in mGPS between the two populations may therefore reflect clinical and tumour characteristics presently unaccounted for; for example obesity and lifestyle factors are important determinants of elevated systemic inflammatory responses and were not measured.^18^^–^20 In addition, ASA grade is relatively subjective and may not fully account for patient co-morbidity,^21^ particularly when compared across different populations and healthcare systems. Furthermore, although speculative, it has recently been suggested that a proportion of patients with mismatch repair (MMR)-deficient colorectal cancer may exhibit elevated systemic inflammatory responses; 22^,^^23^ however given that only a small proportion of tumours arise through MMR deficiency, this may only account for some of the observed differences. It is clear that further characterisation of both tumour and host immune responses is required to fully determine the nature of any differences in systemic inflammatory responses between different populations.

Despite these observed differences, the mGPS showed comparable prognostic value in both cohorts. This would further ratify the mGPS as an inexpensive, readily measured and internationally applicable prognostic marker in patients undergoing surgery for colorectal cancer. Indeed, given the population-based differences in the prevalence of an elevated mGPS, as described in both this study and previous work,^11^ it is clear that such measures should be routinely adopted if outcomes are to be compared globally, particularly in the context of future clinical trials.

Emergency presentation is recognised as a predictor of poor survival of patients undergoing colorectal cancer resection.^24^ Consistent with prior work,^6^^,^^25^ emergency presentation was a determinant of the pre-operative systemic inflammatory response. Of interest, although approximately one-third of patients undergoing emergency surgery were not inflamed, this was not reflected by improved survival of this subgroup. This may reflect the heterogeneous nature of emergency patients, whereby some patients may be acutely inflamed due to an acute event such as perforation or obstruction, thereby necessitating emergency presentation and resection. Further work, detailing the nature of the systemic inflammatory response in this population is merited.

Although limited by a small number of patients in each subgroup, it was of interest that the association between mGPS and survival in patients receiving adjuvant therapy for stage III colon cancer appeared to differ with chemotherapy regime. Whereas an elevated mGPS was associated with poorer survival of patients receiving single-agent 5-FU-based chemotherapy, this was not apparent for those receiving combination therapy with oxaliplatin. The reason for this may simply reflect bias in the selection of patients for different chemotherapy regimens, with older more co-morbid patients more likely to receive single-agent therapy in this cohort. However, it has previously been surmised that systemically inflamed patients may be less likely to complete adjuvant chemotherapy due to increased toxicity.^9^ Therefore future studies of the relationship between systemic inflammation, adjuvant therapy use and outcome are warranted.

The selection of patients with stage II disease who may benefit from adjuvant chemotherapy remains unclear, with current decision-making determined by the presence of high-risk pathologic criteria.^26^ Whether the mGPS may aid in identifying patients likely to benefit would be of considerable interest. In the present study, only 68 patients with stage II colon cancer received adjuvant therapy. Given the wide heterogeneity of tumour pathologic characteristics within this population, meaningful statistical analysis is precluded. Future studies of adjuvant therapy in stage II disease, incorporating measures of the systemic inflammatory response alongside more established markers of high-risk disease are warranted.

The present study is limited by its use of overall survival as the primary endpoint for 3-year survival analysis. However, it has previously been shown that elevated preoperative CRP is associated with poorer cancer-specific prognosis in patients with colorectal cancer from both centres.^6^^,^^15^ Here, the endpoint of overall survival was chosen to account for potential institutional differences in follow-up protocols and attainment of mortality data. Furthermore, overall survival is a pragmatic measure of relevance to patients, and increasingly recognised as a valuable metric for reporting outcome.^27^ Median follow-up time of survivors differed between the two cohorts included, reflecting a relatively large proportion of patients from Norway included in the last year quartile. However, it would be expected that longer follow-up and an increasing number of events would only strengthen the associations observed in the present study. Pathological staging differed slightly between cohorts, with different TNM editions used during different time periods in the Norwegian cohort. However, previous work has suggested that such a change would account for upstaging to node positive disease in less than 3% of patients, with little implication for prognosis.^28^^,^^29^

In conclusion, the present study represents the largest prospective dataset analysing the systemic inflammatory response as measured by mGPS in operable stage I–III CRC to date. Results further confirm the clinical relevance of assessment of the systemic inflammatory response as a prognostic and potentially predictive marker in patients with stage I–III colorectal cancer. The mGPS may be readily applied to the staging of patients undergoing potentially curative resection and should be considered a mandatory characteristic for reporting not only in routine clinical practice but also in future clinical trials.

## Electronic supplementary material

Below is the link to the electronic supplementary material.

Supplementary material 1 (DOCX 54 kb)

Supplementary material 2 (DOCX 17 kb)

Supplementary material 3 (DOCX 16 kb)

Supplementary material 4 (DOCX 18 kb)

Supplementary material 5 (DOCX 19 kb)

## Data Availability

Anonymised data for this study can be provided on request from the corresponding author.
