# Supplementary material for: Systemic Inflammation and Outcome in 2295 Patients with Stage I–III Colorectal Cancer from Scotland and Norway: First Results from the ScotScan Colorectal Cancer Group
Source: Ann Surg Oncol. 2020 Apr 4;27(8):2784–94. doi: 10.1245/s10434-020-08268-1 (PMC7334267; doi:10.1245/s10434-020-08268-1)
Supplement: Supplementary file 1 — Supplementary material 1 (DOCX 54 kb) [file 10434_2020_8268_MOESM1_ESM.docx]

**Supplementary Figure 1.** Distribution of propensity scores (a) before and (b) after matching

a


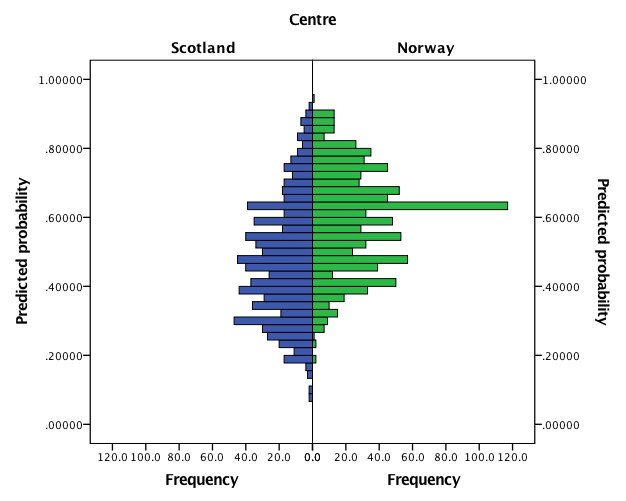


b


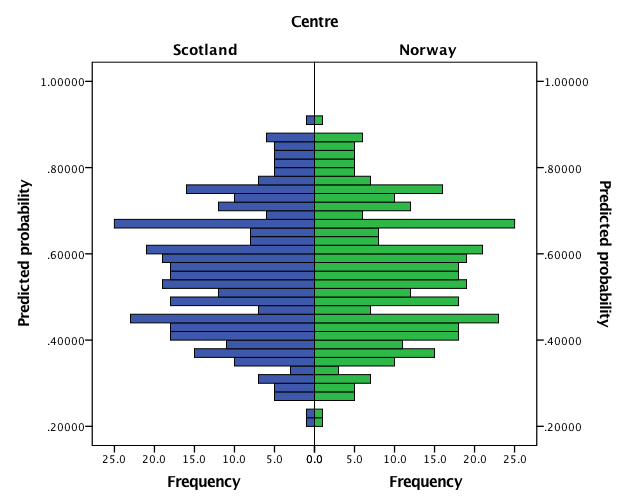


Propensity scoring – age, ASA, presentation, neoadjuvant, adjuvant, location, T stage, N stage, differentiation
