# Supplementary material for: Systemic Inflammation and Outcome in 2295 Patients with Stage I–III Colorectal Cancer from Scotland and Norway: First Results from the ScotScan Colorectal Cancer Group
Source: Ann Surg Oncol. 2020 Apr 4;27(8):2784–94. doi: 10.1245/s10434-020-08268-1 (PMC7334267; doi:10.1245/s10434-020-08268-1)
Supplement: Supplementary file 2 — Supplementary material 2 (DOCX 17 kb) [file 10434_2020_8268_MOESM2_ESM.docx]

Supplementary Table 1. Comparison of clinicopathological characteristics of propensity score matched patients from Scotland, and Norway undergoing potentially curative resection of stage I-III colorectal cancer

|  |  | **Scotland** |  | **Norway** |  |
| --- | --- | --- | --- | --- | --- |
| **Clinicopathological Characteristics** | | **(*N*=368) (%)** |  | **(*N*=368) (%)** | ***P*** |
| **Age** | **<65**  **65-74**  **>75** | 95 (26)  131 (36)  142 (38) |  | 95 (26)  131 (36)  142 (38) | - |
| **Sex** | **Female**  **Male** | 171 (47)  197 (54) |  | 182 (50)  186 (51) | 0.417 |
| **ASA grade** | **I**  **II**  **III**  **IV** | 32 (9)  159 (43)  169 (46)  8 (2) |  | 32 (9)  159 (43)  169 (46)  8 (2) | - |
| **Year of surgery quartile** | **1997-2005**  **2006-2010**  **2011-2013**  **2014-2017** | 182 (50)  82 (22)  53 (14)  51 (14) |  | 13 (3)  102 (28)  109 (30)  144 (39) | <0.001 |
| **Presentation** | **Elective**  **Emergency** | 356 (97)  12 (3) |  | 356 (97)  12 (3) | - |
| **Neoadjuvant chemo (radio) therapy** | **No**  **Yes** | 347 (94)  21 (6) |  | 347 (94)  21 (6) | - |
| **Adjuvant chemo therapy** | **No**  **Yes** | 337 (92)  31 (8) |  | 337 (92)  31 (8) | - |
| **Tumor site** | **Colon**  **Rectum** | 284 (77)  84 (23) |  | 284 (77)  84 (23) | - |
| **Tumour subsite** | **Right**  **Left**  **Rectum** | 152 (41)  132 (36)  84 (23) |  | 152 (41)  132 (36)  84 (23) | - |
| **T stage** | **0**  **1**  **2**  **3**  **4** | 1 (0)  28 (8)  63 (17)  253 (69)  23 (6) |  | 1 (0)  28 (8)  63 (17)  253 (69)  23 (6) | - |
| **N stage** | **0**  **1**  **2** | 288 (78)  67 (18)  13 (4) |  | 288 (78)  67 (18)  13 (4) | - |
| **TNM stage** | **PCR**  **I**  **II**  **III** | 1 (0)  87 (24)  200 (54)  80 (22) |  | 1 (0)  87 (24)  200 (54)  80 (22) | - |
| **Differentiation** | **Well/ mod**  **Poor** | 337 (92)  31 (8) |  | 337 (92)  31 (8) | - |
| **C-reactive protein** | **≤10mg/L**  **>10mg/L** | 241 (65)  127 (35) |  | 310 (84)  58 (16) | <0.001 |
| **Albumin** | **≥35g/L**  **<35g/L** | 269 (73)  99 (27) |  | 353 (96)  15 (4) | <0.001 |
| **mGPS** | **0**  **1**  **2** | 241 (65)  69 (19)  58 (16) |  | 310 (84)  45 (12)  13 (3) | <0.001 |
