# Supplementary material for: Systemic Inflammation and Outcome in 2295 Patients with Stage I–III Colorectal Cancer from Scotland and Norway: First Results from the ScotScan Colorectal Cancer Group
Source: Ann Surg Oncol. 2020 Apr 4;27(8):2784–94. doi: 10.1245/s10434-020-08268-1 (PMC7334267; doi:10.1245/s10434-020-08268-1)
Supplement: Supplementary file 3 — Supplementary material 3 (DOCX 16 kb) [file 10434_2020_8268_MOESM3_ESM.docx]

Supplementary Table 2. Comparison of clinicopathological characteristics of patients from Scotland and Norway undergoing potentially curative resection of stage I-III colon cancer without neoadjuvant therapy

|  |  | **Scotland** |  | **Norway** |  |
| --- | --- | --- | --- | --- | --- |
| **Clinicopathological Characteristics** | | **(*N*=823) (%)** |  | **(*N*=795) (%)** | ***P*** |
| **Age** | **<65**  **65-74**  **>75** | 256 (31)  279 (34)  288 (35) |  | 153 (19)  243 (31)  300 (50) | <0.001 |
| **Sex** | **Female**  **Male** | 376 (46)  447 (54) |  | 450 (57)  345 (43) | <0.001 |
| **ASA grade (1346)** | **I**  **II**  **III**  **IV** | 100 (17)  246 (41)  219 (37)  31 (5) |  | 31 (4)  286 (38)  394 (53)  39 (5) | <0.001 |
| **Presentation (1617)** | **Elective**  **Emergency** | 712 (86)  111 (14) |  | 706 (89)  88 (11) | 0.141 |
| **Adjuvant therapy (1588)** | **No**  **Yes** | 586 (74)  207 (26) |  | 642 (81)  153 (19) | 0.001 |
| **Tumour subsite** | **Right colon**  **Left colon** | 448 (55)  369 (45) |  | 460 (58)  335 (42) | 0.221 |
| **T stage** | **0**  **1**  **2**  **3**  **4** | 0 (0)  56 (7)  80 (10)  432 (52)  255 (31) |  | 1 (0)  62 (8)  117 (15)  563 (71)  52 (6) | <0.001 |
| **N stage** | **0**  **1**  **2** | 512 (62)  219 (27)  92 (11) |  | 523 (66)  204 (26)  68 (8) | 0.062 |
| **TNM stage** | **0**  **I**  **II**  **III** | 0 (0)  120 (15)  392 (47)  311 (38) |  | 1 (0)  153 (19)  369 (46)  272 (34) | 0.015 |
| **Differentiation (1562)** | **Well/ mod**  **Poor** | 730 (89)  86 (11) |  | 614 (82)  132 (18) | <0.001 |
| **C-reactive protein** | **≤10mg/L**  **>10mg/L** | 494 (60)  329 (40) |  | 522 (66)  273 (34) | 0.019 |
| **Albumin** | **≥35g/L**  **<35g/L** | 572 (69)  251 (31) |  | 686 (86)  109 (14) | <0.001 |
| **mGPS** | **0**  **1**  **2** | 494 (60)  174 (21)  155 (19) |  | 522 (66)  183 (23)  90 (11) | <0.001 |

(*n*) given when incomplete data available. *P*-value given for χ^2^ method for linear trend.
