# Supplementary material for: Systemic Inflammation and Outcome in 2295 Patients with Stage I–III Colorectal Cancer from Scotland and Norway: First Results from the ScotScan Colorectal Cancer Group
Source: Ann Surg Oncol. 2020 Apr 4;27(8):2784–94. doi: 10.1245/s10434-020-08268-1 (PMC7334267; doi:10.1245/s10434-020-08268-1)
Supplement: Supplementary file 4 — Supplementary material 4 (DOCX 18 kb) [file 10434_2020_8268_MOESM4_ESM.docx]

**Supplementary Table 3.** Relationship between clinicopathological characteristics and overall survival of patients undergoing potentially curative resection of stage I-III colorectal cancer in Norway and Scotland (*n*=2295)

|  |  | Univariate HR  (95% CI) | *P* |  | Multivariate HR (95% CI) | *P* |
| --- | --- | --- | --- | --- | --- | --- |
| Scotland | | | | | | |
| Age (<65/ 65-74/ >74) |  | 1.75 (1.57-1.95) | <0.001 |  | 1.47 (1.28-1.69) | <0.001 |
| Sex (female/ male) |  | 1.16 (0.98-1.37) | 0.080 |  | 1.23 (1.01-1.51) | 0.045 |
| ASA grade (I/ II/ III/ IV) |  | 1.89 (1.65-2.16) | <0.001 |  | 1.55 (1.34-1.79) | <0.001 |
| Presentation (elective/ emergency) |  | 1.51 (1.16-1.96) | 0.002 |  | - | 0.429 |
| Year of surgery quartile |  | 0.82 (0.75-0.91) | <0.001 |  | - | 0.581 |
| Neoadjuvant therapy (No/ yes) |  | 0.78 (0.57-1.07) | 0.121 |  | - | - |
| Adjuvant therapy (No/ yes) |  | 0.74 (0.60-0.91) | 0.004 |  | 0.70 (0.53-0.93) | 0.012 |
| Tumour site (Right/ left/ rectum) |  | 0.93 (0.85-1.03) | 0.182 |  | - | - |
| T stage (0/ 1/ 2/ 3/ 4) |  | 1.47 (1.31-1.64) | <0.001 |  | 1.30 (1.12-1.51) | 0.001 |
| N stage (0/ 1/ 2) |  | 1.40 (1.25-1.57) | <0.001 |  | 1.45 (1.25-1.68) | <0.001 |
| Differentiation (Mod-well/ poor) |  | 1.49 (1.15-1.93) | 0.003 |  | - | 0.165 |
| Modified Glasgow Prognostic Score (0/ 1/ 2) |  | 1.45 (1.31-1.61) | <0.001 |  | 1.27 (1.12-1.45) | <0.001 |
| Norway | | | | | | |
| Age (<65/ 65-74/ >74) |  | 2.50 (2.03-3.08) | <0.001 |  | 1.86 (1.43-2.41) | <0.001 |
| Sex (female/ male) |  | 0.99 (0.77-1.29) | 0.957 |  | - | - |
| ASA grade (I/ II/ III/ IV) |  | 3.69 (2.91-4.69) | <0.001 |  | 2.71 (2.04-3.60) | <0.001 |
| Presentation (elective/ emergency) |  | 2.56 (1.81-3.61) | <0.001 |  | 1.85 (1.20-2.86) | 0.005 |
| Year of surgery quartile |  | 0.68 (0.58-0.79) | <0.001 |  | - | 0.078 |
| Neoadjuvant therapy (No/ yes) |  | 0.43 (0.24-0.79) | 0.006 |  | - | 0.574 |
| Adjuvant therapy (No/ yes) |  | 0.54 (0.35-0.85) | 0.008 |  | - | 0.116 |
| Tumour site (Right/ left/ rectum) |  | 0.79 (0.67-0.93) | 0.005 |  | - | 0.343 |
| T stage (0/ 1/ 2/ 3/ 4) |  | 1.60 (1.30-1.96) | <0.001 |  | - | 0.255 |
| N stage (0/ 1/ 2) |  | 1.44 (1.19-1.74) | <0.001 |  | 1.39 (1.11-1.73) | 0.004 |
| Differentiation (Mod-well/ poor) |  | 1.31 (0.93-1.85) | 0.123 |  | - | 0.739 |
| Modified Glasgow Prognostic Score (0/ 1/ 2) |  | 1.86 (1.59-2.18) | <0.001 |  | 1.23 (1.01-1.49) | 0.043 |

HR – hazard ratio, 95%CI – 95% confidence interval, ASA – American Society of Anesthesiologists
