# Supplementary material for: Systemic Inflammation and Outcome in 2295 Patients with Stage I–III Colorectal Cancer from Scotland and Norway: First Results from the ScotScan Colorectal Cancer Group
Source: Ann Surg Oncol. 2020 Apr 4;27(8):2784–94. doi: 10.1245/s10434-020-08268-1 (PMC7334267; doi:10.1245/s10434-020-08268-1)
Supplement: Supplementary file 5 — Supplementary material 5 (DOCX 19 kb) [file 10434_2020_8268_MOESM5_ESM.docx]

Supplementary Table 4. Relationship between systemic inflammatory response and clinicopathological characteristics according to adjuvant chemotherapy regime in patients with stage III colon cancer

|  | | **No chemotherapy** | | | | |  | **5-FU alone** | | | | |  | **Oxaliplatin combined** | | | | |
| --- | --- | --- | --- | --- | --- | --- | --- | --- | --- | --- | --- | --- | --- | --- | --- | --- | --- | --- |
|  | | **CRP≤10mg/L** |  | **CRP>10mg/L** |  | ***P*** |  | **CRP≤10mg/L** |  | **CRP>10mg/L** |  | ***P*** |  | **CRP≤10mg/L** |  | **CRP>10mg/L** |  | ***P*** |
| **Age** | **<65**  **65-74**  **>75** | 22 (15)  30 (21)  93 (64) |  | 11 (9)  20 (17)  86 (74) |  | 0.085 |  | 7 (16)  24 (56)  12 (28) |  | 2 (7)  18 (62)  9 (31) |  | 0.474 |  | 64 (60)  42 (39)  1 (1) |  | 23 (56)  17 (42)  1 (2) |  | 0.587 |
| **Sex** | **Female**  **Male** | 74 (51)  71 (49) |  | 68 (58)  49 (42) |  | 0.253 |  | 18 (42)  25 (58) |  | 17 (59)  12 (41) |  | 0.166 |  | 51 (48)  56 (52) |  | 26 (63)  15 (37) |  | 0.087 |
| **Location** | **Scotland**  **Norway** | 80 (55)  65 (45) |  | 71 (61)  46 (39) |  | 0.370 |  | 8 (19)  35 (81) |  | 3 (10)  26 (90) |  | 0.343 |  | 46 (43)  61 (57) |  | 17 (42)  24 (58) |  | 0.867 |
| **ASA grade** (399) | **I**  **II**  **III**  **IV** | 6 (5)  30 (24)  75 (61)  12 (10) |  | 3 (3)  20 (22)  54 (59)  14 (15) |  | 0.246 |  | 3 (8)  10 (26)  25 (66)  0 (0) |  | 2 (8)  10 (42)  12 (50)  0 (0) |  | 0.336 |  | 21 (23)  57 (63)  12 (13)  1 (1) |  | 4 (13)  20 (62)  8 (25)  0 (0) |  | 0.121 |
| **Year of surgery quartile** | **1997-2005**  **2006-2010**  **2011-2013**  **2014-2017** | 44 (30)  37 (25)  24 (17)  40 (28) |  | 44 (38)  34 (29)  18 (15)  21 (18) |  | 0.055 |  | 2 (5)  6 (14)  13 (30)  22 (51) |  | 2 (7)  11 (38)  8 (27)  8 (27) |  | 0.021 |  | 0 (0)  22 (21)  41 (38)  44 (41) |  | 1 (2)  8 (20)  13 (32)  19 (46) |  | 0.923 |
| **Presentation** | **Elective**  **Emergency** | 130 (90)  15 (10) |  | 89 (76)  28 (24) |  | 0.003 |  | 38 (88)  5 (12) |  | 22 (76)  7 (24) |  | 0.165 |  | 99 (92)  8 (8) |  | 27 (66)  14 (34) |  | <0.001 |
| **Tumour subsite** (478) | **Right**  **Left** | 83 (58)  61 (42) |  | 74 (64)  42 (36) |  | 0.314 |  | 16 (37)  27 (63) |  | 13 (45)  16 (55) |  | 0.521 |  | 53 (52)  52 (49) |  | 26 (63)  15 (37) |  | 0.062 |
| **T stage** | **1**  **2**  **3**  **4** | 0 (0)  8 (5)  97 (67)  40 (28) |  | 2 (2)  2 (2)  65 (55)  48 (41) |  | 0.051 |  | 2 (5)  5 (11)  34 (79)  2 (5) |  | 1 (3)  0 (0)  20 (69)  8 (28) |  | 0.013 |  | 4 (4)  14 (13)  71 (66)  18 (17) |  | 0 (0)  0 (0)  26 (63)  15 (37) |  | 0.001 |
| **N stage** | **1**  **2** | 118 (81)  27 (19) |  | 78 (67)  39 (33) |  | 0.006 |  | 34 (79)  9 (21) |  | 21 (72)  8 (28) |  | 0.517 |  | 73 (68)  34 (32) |  | 25 (61)  16 (39) |  | 0.406 |
| **Differentiation** (467) | **Well/ mod**  **Poor** | 125 (88)  17 (12) |  | 89 (77)  26 (23) |  | 0.023 |  | 35 (85)  6 (15) |  | 21 (72)  8 (28) |  | 0.185 |  | 95 (92)  8 (8) |  | 24 (65)  13 (35) |  | <0.001 |

(*n*) given when incomplete data available. *P*-value given for χ^2^ method for linear trend.
